# Are we prematurely predicting acute mountain sickness?

**DOI:** 10.1113/EP092490

**Published:** 2025-01-20

**Authors:** Julian C. Bommarito, Michael M. Tymko

**Affiliations:** ^1^ Human Cardiovascular Physiology Laboratory University of Guelph Guelph Ontario Canada; ^2^ Integrative Cerebrovascular and Environmental Physiology SB Laboratory University of Guelph Guelph Ontario Canada

**Keywords:** acute mountain sickness, high altitude, nocturnal oxygen saturation, preterm birth

1

Acute mountain sickness (AMS) commonly affects individuals ascending to altitudes above ∼2500 m, and is characterized by the presence of headache, along with other symptoms such as gastrointestinal distress, dizziness/light‐headedness, and general fatigue and weakness. Healthy adults born preterm (<37 weeks of gestation) may be at greater risk for AMS due to differences in pulmonary function and respiratory loop gain (Bates et al., 2014) and have a higher prevalence of sleep‐disordered breathing compared to their term born healthy counterparts (Crump et al., 2019).

Predicting the occurrence of AMS in individuals is often regarded as the ‘holy grail’ of high‐altitude research. Among the potential predictors, nocturnal $S_{pO_{2}}$ stands out as the most promising. A recent study by Joyce et al. (2024) tested 18 healthy adults (seven females and 11 males; 36 ± 16 years) ascending to 4800 m altitude over 12 days. AMS was assessed through the Lake Louise Scoring Questionnaire each morning, and nocturnal $S_{pO_{2}}$ was continuously recorded at 3300, 3850 and 4800 m. The authors found that overnight $S_{pO_{2}}$ at 3850 and 4800 m was moderately correlated (*r* ^2^ = 0.34 and 0.43, respectively) with AMS scores the morning after arrival at 4800 m.

In this issue of *Experimental Physiology*, Narang et al. (2025) investigated the relationship between nocturnal $S_{pO_{2}}$ and AMS in individuals born full term versus preterm. In this well‐designed study, the authors recruited 24 young, healthy adult males: 12 born at term and 12 born very preterm (gestational age ≤32 weeks and gestational mass ≤1500 g), matched for age, body mass index, ${\dot{V}}_{O_{2}\mathrm{peak}}$, and pulmonary function (i.e., FVC, FEV_1_, DL_CO_, *K*_CO_ and alveolar volume). Participants were exposed to normobaric hypoxia for ∼16–17 h (equivalent to ∼4200 m). The primary outcome was to compare nocturnal $S_{pO_{2}}$ metrics, which measure the adjustments of oxygen saturation levels overnight (i.e., the mean, minimum and morning $S_{pO_{2}}$, proportion of time $S_{pO_{2}}$ was <80% (TST80), difference in mean $S_{pO_{2}}$ between the two halves of the night (∆$S_{pO_{2}}$), mean $S_{pO_{2}}$ of the final nocturnal hour, number and duration of desaturations, oxygen desaturation index, and hypoxic burden) between full‐term and preterm participant groups during a night in normobaric hypoxia. The secondary outcome was to determine whether nocturnal $S_{pO_{2}}$ metrics predicted the development of AMS.

Contrary to the authors' hypotheses, mean and minimum $S_{pO_{2}}$ levels and the TST80 were not different between groups. However, the preterm group exhibited a lower ∆$S_{pO_{2}}$, and a greater nocturnal desaturation‐induced hypoxic burden, which is calculated as the area under the curve during desaturation events, relative to their respective predesaturation baseline values. Additionally, in the preterm group only, the mean, minimum and morning $S_{pO_{2}}$, along with TST80, predicted morning AMS scores.

The elegant findings presented by Narang et al. (2025) offer several promising directions for future exploration. First, the inclusion of females in future studies to assess potential sex differences in the preterm group. Prior work indicates that periodic breathing is more prominent in males during sleep at altitude compared to females (Lombardi et al., 2013). Whether this sex difference exists among preterm individuals and how it may influence AMS development remains unclear. Second, assessing the interaction between chemosensitivity and AMS would provide mechanistic insight into AMS. Adults born preterm are a particularly interesting population for research to assess this interaction due to their blunted hypoxic ventilatory response (Bates et al., 2014). Lastly, the observed elevation (*P* = 0.05) in nocturnal heart rate in the preterm group deserves further exploration. Speculating on why nocturnal heart rate differed between groups is difficult without assessing nocturnal heart rate in normoxia (i.e., having a baseline condition). Measuring nocturnal cardiac baroreflex sensitivity and blood pressure variability between full‐term and preterm born individuals could provide a unique opportunity in potential cardiovascular regulation differences between these two populations. Notably, aortic stiffness is elevated in preterm born adults compared to their full‐term born counterparts (Barnard et al., 2020), which may indicate reduced baroreflex sensitivity and heightened blood pressure variability.

AMS can range from being slightly uncomfortable to, in extreme circumstances, life threatening when it progresses to more serious altitude‐related illnesses such as high‐altitude pulmonary oedema or high‐altitude cerebral oedema. Understanding the risk of developing AMS across different populations is essential to fully understand the underlying mechanism(s). For example, the effects of a commonly used pharmacological treatment for AMS, acetazolamide, is completely understudied in preterm birth, along with new and promising non‐pharmacological treatments such as exogenous ketone supplementation.

Narang et al. (2025) observed that mean and minimum $S_{pO_{2}}$ levels and the TST80 did not differ between groups, but nocturnal $S_{pO_{2}}$ recovery was lower, and the relative hypoxic burden was greater in the preterm group. Interestingly, the authors identified the predictive capacity of composite $S_{pO_{2}}$ variables on AMS such as mean, minimum and morning $S_{pO_{2}}$ and TST80 in the preterm group only, but reasoning as to why this was not observed in full‐term birth is unclear, though it might be due to methodological differences from Joyce et al. (2024), who conducted a field research expedition rather than an acute hypoxia study in normobaria. Nevertheless, the results presented by Narang et al. (2025) are exciting as they underscore the potential utility of nocturnal $S_{pO_{2}}$ metrics, particularly in individuals born preterm, for predicting AMS risk. These findings highlight the importance of considering birth history (in lowlander and highlander populations) in high‐altitude research.

## AUTHOR CONTRIBUTIONS

Both authors have read and approved the final version of this manuscript and agree to be accountable for all aspects of the work in ensuring that questions related to the accuracy or integrity of any part of the work are appropriately investigated and resolved. All persons designated as authors qualify for authorship, and all those who qualify for authorship are listed.

## CONFLICT OF INTEREST

None declared.

## References

[eph13759-bib-0001] Barnard, C. R. , Peters, M. , Sindler, A. L. , Farrell, E. T. , Baker, K. R. , Palta, M. , Stauss, H. M. , Dagle, J. M. , Segar, J. , Pierce, G. L. , Eldridge, M. W. , & Bates, M. L. (2020). Increased aortic stiffness and elevated blood pressure in response to exercise in adult survivors of prematurity. Physiological Reports, 8(12), e14462. 32562387 10.14814/phy2.14462 PMC7305240

[eph13759-bib-0002] Bates, M. L. , Farrell, E. T. , & Eldridge, M. W. (2014). Abnormal ventilatory responses in adults born prematurely. New England Journal of Medicine, 370(6), 584–585. 24499235 10.1056/NEJMc1311092 PMC4769592

[eph13759-bib-0003] Crump, C. , Friberg, D. , Li, X. , Sundquist, J. , & Sundquist, K. (2019). Preterm birth and risk of sleep‐disordered breathing from childhood into mid‐adulthood. International Journal of Epidemiology, 48(6), 2039–2049. 31006012 10.1093/ije/dyz075 PMC6929528

[eph13759-bib-0004] Joyce, K. E. , Ashdown, K. , Delamere, J. P. , Bradley, C. , Lewis, C. T. , Letchford, A. , Lucas, R. A. I. , Malein, W. , Thomas, O. , Bradwell, A. R. , & Lucas, S. J. E. (2024). Nocturnal pulse oximetry for the detection and prediction of acute mountain sickness: An observational study. Experimental Physiology, 109(11), 1856–1868. 39277825 10.1113/EP091691 PMC11522851

[eph13759-bib-0005] Lombardi, C. , Meriggi, P. , Agostoni, P. , Faini, A. , Bilo, G. , Revera, M. , Caldara, G. , Di Rienzo, M. , Castiglioni, P. , Maurizio, B. , Gregorini, F. , Mancia, G. , & Parati, G. , & The Highcare Investigators . (2013). High‐altitude hypoxia and periodic breathing during sleep: Gender‐related differences. Journal of Sleep Research, 22(3), 322–330. 23294420 10.1111/jsr.12012

[eph13759-bib-0006] Narang, B. J. , Manferdelli, G. , Millet, G. P. , & Debevec, T. (2025). Nocturnal pulse oxygen saturation dynamics at simulated high altitude: Predictive value for acute mountain sickness in healthy men born pre‐term. Experimental Physiology, 110(6), 821–831. 10.1113/EP092418 PMC12128470 39817525

